# 948 CPS Reporting Outcomes of Pediatric Burns

**DOI:** 10.1093/jbcr/iraf019.479

**Published:** 2025-04-01

**Authors:** Michael Yu, Camsy Huang, Suzanne Dakil, Rebecca Coffey, Chiaka Akarichi

**Affiliations:** University of Texas Southwestern, Parkland Burn Center; University of Texas Southwestern Medical School; University of Texas Southwestern; University of Texas Southwestern, Parkland Burn Center; University of Texas Southwestern, Parkland Burn Center

## Abstract

**Introduction:**

Burns disproportionately affect children, especially younger children. 6-20% of physically abused children have sustained burns1, but it is often difficult to differentiate between accidental and abusive pediatric burns. Over 60% of pediatric burns occur in children under 5 years old, an age where an accurate history is difficult to obtain. In cases of abuse and neglect, there is ongoing conversation regarding reporting to Child Protective Services (CPS). CPS referrals can be placed at tertiary burn centers where there is a child abuse team to evaluate for suspicions of maltreatment or from outside community hospitals who do not regularly evaluate burns or pediatric patients. Prior research has evaluated the changes regarding the diagnosis of physical abuse when presented to a non-child abuse physician versus a child abuse pediatrician (Anderst, 2009). To our knowledge, there are no studies evaluating reporting rates and CPS outcomes for burns that are concerning for child abuse and neglect (maltreatment).

**Methods:**

The study is a case control chart review comparing all pediatric burns (patients 18 and under) at a verified burn center between 2018-2022. The burn center data registry was analyzed for significant results using Chi square analysis. Comparisons between CPS reports versus no CPS reports and differences between CPS response and no CPS response were analyzed. Demographic and bur specific information was also collected.

**Results:**

From 2018-2022, there were 1017 admitted pediatric patients. There were 101 CPS reports from the outside hospital resulting in 7 CPS removal (6.9%). The child abuse team was consulted in 82 cases (81%) and 34 cases had concerns of maltreatment (41.4%). There were 88 CPS reports from the burn center resulting in 9 CPS removal (10.2%). The child abuse team was consulted in 69 cases (78%) and 31 cases had concerns of maltreatment (44.9%). There was no statistical significance in CPS removal (Chi square 0.65, p value 0.42) or in determination for maltreatment (Chi-square 0.18, p value 0.67) based on reporting from the referring hospital and burn center. There were 52 cases where CPS was involved that the child abuse team was not consulted, which represents 24% of all CPS cases in the five year period. The child abuse team was able to prevent 50 cases at the burn center from leading to a potentially unnecessary CPS report.

**Conclusions:**

While there was no statistical significance between CPS reporting outcomes or determination of maltreatment between the referring hospital and the burn center, the importance of consulting those with expertise in child abuse and neglect is paramount to ensuring the child’s safety and giving caregivers the resource and support they need.

**Applicability of Research to Practice:**

Further research into reporting bias (age, race, insurance status, type of burn, total burn surface area, etc.) and resource utilization is planned.

**Funding for the Study:**

N/A

